# The Profile of Heparanase Expression Distinguishes Differentiated Thyroid Carcinoma from Benign Neoplasms

**DOI:** 10.1371/journal.pone.0141139

**Published:** 2015-10-21

**Authors:** Leandro Luongo Matos, Eloah Rabello Suarez, Thérèse Rachell Theodoro, Damila Cristina Trufelli, Carina Mucciolo Melo, Larissa Ferraz Garcia, Olivia Capela Grimaldi Oliveira, Maria Graciela Luongo Matos, Jossi Ledo Kanda, Helena Bonciani Nader, João Roberto Maciel Martins, Maria Aparecida Silva Pinhal

**Affiliations:** 1 Biochemistry Department, Faculdade de Medicina do ABC, Santo André, Brazil; 2 Head and Neck Surgery Department, Faculdade de Medicina do ABC, Santo André, Brazil; 3 Biochemistry Department, Universidade Federal de São Paulo, São Paulo, Brazil; 4 Laboratory of Molecular and Translational Endocrinology, Endocrinology and Metabolism Discipline, Universidade Federal de São Paulo, São Paulo, Brazil; 5 Pathology Department, Pathos Medical Diagnosis, São Paulo, Brazil; 6 Universidade Cidade de São Paulo (Unicid), São Paulo, Brazil; University of Patras, GREECE

## Abstract

**Introduction:**

The search for a specific marker that could help to distinguish between differentiated thyroid carcinoma and benign lesions remains elusive in clinical practice. Heparanase (HPSE) is an endo-beta-glucoronidase implicated in the process of tumor invasion, and the heparanase-2 (HPSE2) modulates HPSE activity. The aim of this study was to evaluate the role of heparanases in the development and differential diagnosis of follicular pattern thyroid lesions.

**Methods:**

HPSE and HPSE2 expression by qRT-PCR, immunohistochemistry evaluation, western blot analysis and HPSE enzymatic activity were evaluated.

**Results:**

The expression of heparanases by qRT-PCR showed an increase of HPSE2 in thyroid carcinoma (P = 0.001). HPSE activity was found to be higher in the malignant neoplasms than in the benign tumors (P<0.0001). On Western blot analysis, HPSE2 isoforms were detected only in malignant tumors. The immunohistochemical assay allowed us to establish a distinct pattern for malignant and benign tumors. Carcinomas showed a typical combination of positive labeling for neoplastic cells and negative immunostaining in colloid, when compared to benign tumors (P<0.0001). The proposed diagnostic test presents sensitivity and negative predictive value of around 100%, showing itself to be an accurate test for distinguishing between malignant and benign lesions.

**Conclusions:**

This study shows, for the first time, a distinct profile of HPSE expression in thyroid carcinoma suggesting its role in carcinogenesis.

## Introduction

Thyroid nodules are very common in the general population and are usually benign (85%-95%).[1, 2] Ultrasound-guided fine-needle aspiration (FNAB) is the best established method for thyroid nodule evaluation.[3] However, a significant percentage of this cytology is classified as “indeterminate” and the rates of malignancy are very broad, ranging from 10% to 30%. The majority of these patients are submitted to a theoretically diagnostic thyroidectomy. In the last few years, several protein and genetic markers have been employed to distinguish between benign and malignant lesions in order to improve the diagnosis of FNAB. For instance, immunological studies with several markers have been performed but the results and applications of these markers are still controversial.[4–6] Indeed, these molecules have not been proven to have the specificity and, more critically, enough sensitivity in the differentiation of follicular lesions, besides the remaining variable rates of false-negative results.[7, 8]

In addition several extracellular matrix components of tumor-associated stromal cells might influence the growth and progression of most human carcinomas and, thus, could contribute either as diagnostic or therapeutic tools [9]. One of these components is heparanase, an endo-beta-glucuronidase, which is known to promote the progression of many cancers due to enzymatic degradation of heparan sulfate (HS) that can liberate heparin-binding growth factors and remodel the extracellular matrix to facilitate tumor invasiveness and metastasis.[10–12] So far, the involvement of heparanase/heparan sulfate in thyroid tumorigenesis has been scarcely reported.[13, 14] There are two heparanase family members, heparanase (HPSE) and heparanase-2 (HPSE2). [15] HPSE has been found in two forms: one presenting 65 kDa and described as a precursor with no apparent enzymatic activity and the other a mature active enzyme, a heterodimer with a 50 kDa C-terminal subunit, resulting from protease processing, and an 8-kDa N-terminal subunit.[16, 17] HPSE2 has three alternative variant splice transcripts, HPSE2a, b and c, which encode putative proteins of 480, 534, and 592 amino acids, respectively, and shares an overall similarity of 35% with HPSE.[15] Studies do not clarify the contribution of HPSE2 in human carcinogenesis, since it does not present enzymatic activity as HPSE.[15, 18]

Therefore, the aim of the present study was to study the role of heparanase and heparanase-2 in thyroid carcinogenesis, in an effort to contribute to distinguishing between differentiated thyroid carcinoma and benign lesions.

## Material and Methods

The research was performed using two studies, one prospective, in order to evaluate heparanase biology in normal thyroid and also in malignant and benign neoplasms; and the other retrospective, to analyze heparanase expression as a diagnostic test to distinguish between differentiated thyroid carcinoma (DTC) and benign lesions.

### Prospective sample

A total of 27 surgically obtained thyroid samples were selected from patients submitted to thyroidectomy (24 women and 3 men with mean age of 57 ± 11 years) indicated by cytologically indeterminate FNAB (Bethesda system III-V) in the year of 2010. The histopathological examination of these samples revealed: 15 DTC (4 follicular variant of papillary carcinomas = FVPTC; 9 classic papillary carcinomas = PTC; and 2 follicular carcinomas = FC) and 12 benign lesions (2 follicular adenomas = FA; 7 hyperplastic nodules = HN; and 3 Hashimoto’s thyroiditis = HT). Adjacent thyroid tissues, presented in 22 cases, were also analyzed. The tissue specimens were preserved in both RNA stabilizer (RNA Holder®, São Paulo, SP, Brazil) and Tissue-Tek O.C.T. Compound (Sakura Finetek®, Alphen aan den Rijn, Holland) and stored at -80°C. Other fragments were formalin-fixed and paraffin-embedded for immunological analysis. Six cases (2 PTC, and 4 benign lesions) of FNAB specimens were than obtained to test HPSE2 as a preliminary diagnostic study.

### Retrospective sample

230 specimens were selected from the archives of the Pathology Department. Sixty-one DTC samples (33 PTC, 15 FVPTC and 13 FC) and 169 benign lesions (60 FA, 49 HN and 60 nodular goiters = NG) were used, aiming at a 1:3 case-control study. Adjacent normal thyroid tissue was also analyzed in 221 cases. No additional clinicopathological parameters were recovered for HPSE/HPSE2 correlations since the aim of the study was to investigate these proteins as a diagnostic tool.

### Ethics

The research protocol was approved by the Research Ethics Committees of the Faculdade de Medicina do ABC (protocol: 098/2008) and the Universidade Federal de São Paulo (protocol: 086/2007). All patients included were informed about the study and signed an informed consent.

### Immunocyto- and Immunohistochemistry

Three-micrometer-thick sections of formalin-fixed paraffin-embedded tissues were deparaffinized and rehydrated. The primary heparanase antibodies, HPA1 H-80 (product sc25825; Santa Cruz®, Biotechnology, CA, USA) and HPA2 C-17 (product sc14900; Santa Cruz®, Biotechnology, CA, USA), were diluted 1:100 in bovine serum albumin and incubated overnight. Universal secondary biotinylated antibody (LSAB®, DakoCytomation®, Glostrup, Denmark) was incubated for 30 minutes, and the slides were subsequently developed with a peroxidase-labeled streptavidin complex (LSAB®, DakoCytomation®, Glostrup, Denmark) for an additional 30 minutes. The sections were revealed using 3,3’-diaminobenzidine (DakoCytomation®, Glostrup, Denmark) as the chromogen for one minute and were subsequently counterstained with hematoxylin. Evidence of positive expression was considered to be brownish color (positive staining) and at least mild staining (greater than or equal to ++/++++) of over 10% of cells and over 50% of all colloid areas. Histological slices of pulmonary carcinoid tumor previously tested as positive for heparanase staining were used as positive reaction control and also for negative controls with the omission of the primary antibody and stained only with secondary antibody. Unstained red blood cells and labeled foamy cells were considered, respectively, as negative and positive internal controls. In order to test the specificity of HPSE2 immunohistochemistry reaction, a blocking assay was performed combining the antibody HPA2 C-17 (product sc14900; Santa Cruz®, Biotechnology, CA, USA) at 2 μg/mL with five-fold excess (10 μg/mL) of blocking peptide HPA2 C-17 (product sc14900P; Santa Cruz®, Biotechnology, CA, USA) incubated overnight at 4°C followed by immunohistochemistry reaction as described above. This assay demonstrated the specificity of HPA2 C-17 antibody for the antigen, since there was no immunostaining when the antibody and blocking peptide were used together, as demonstrated in S1 Fig.

### mRNA extraction and quantitative RT-PCR analysis

Total RNA was extracted from thyroid tissue resections using an RNAspin Mini Kit (GE Healthcare®, Munich, Germany). RNA quantification was performed using an Agilent 2100 Bioanalyzer with an RNA 6000 Nano LabChip Kit (Agilent Technologies®, Palo Alto, CA, USA), and the RNA integrity was analyzed via agarose gel electrophoresis to identify the 28S and 18S ribosomal rRNA. The first-strand cDNA obtained was synthesized using 5 μg of total RNA, 500 ng of oligo (dT) and Superscript III reverse transcriptase (Invitrogen®, Carlsbad, USA). The reaction was performed at 50°C for 60 minutes followed by a 15 minutes incubation period at 70°C. Quantitative RT-PCR was performed using SYBR Green I according to the manufacturer’s instructions (Applied Biosystems®, Foster City, CA, USA) at Rotor-Gene 6000 Series Software 1.7 (Corbett Research®, Foster City, CA, USA). The relative expression levels were measured using an efficiency correction, which considers the differences in primer-pair amplification efficiencies between the target and reference genes and results in a more reliable estimation of the ‘‘real expression ratio” than the 2^-ΔCt^ method, and the ΔCt value describes the difference between the CT (cycle threshold) of the target gene and the corresponding endogenous reference gene (housekeeping gene). Measurements of glyceraldehyde-3-phosphate dehydrogenase (GAPDH) and ribosomal protein RPL13a gene expression were used as endogenous controls, the values for sample amplification were obtained using the geometric average of both control genes. The PCR amplifications were performed using the following primer sequences, used previously in other studies by our group[19, 20]: HPSE forward primer 5’-TGGCAAGAAGGTCTGGTTAGGAGA-3’; HPSE reverse primer 5’-GCAAAGGTGTCGGATAGCAAGGG-3’; HPSE2 forward primer 5’-CGCCTGTTAGACACACTCTCTGA-3’; HPSE2 reverse primer 5’-GTCACCACACCTTCAAGCCAA-3’; GAPDH forward primer 5’-GGAGAAGGCTGGGGCTC-3’; GAPDH reverse primer 5’-GTCCTTCCACGATACCAAAG-3’; the ribosomal protein (RPL13a) forward primer 5’-TTGAGGACCTCTGTGTATTTGTCAA-3’; and the RPL13a reverse primer 5’-CCTGGAGGAGAAGAGGAAAGAGA-3’. The post-amplification melting curve analysis was performed to confirm whether the nonspecific amplification was generated from primer-dimers. The assays were performed in triplicate and the experiments were performed twice.

### Heparanase enzymatic assay

An enzymatic assay was carried out as previously described with slight modification[21]. Briefly, biotinylated HS, prepared in our laboratory[22, 23], was dissolved in coating buffer (0.06M NaHCO3, pH 9.6) and uniformly added on microwell plates (FluoroNUNC Maxisorp-96 wells, Roskilde, Denmark). Tissues fragments were weighed and macerated using liquid nitrogen and then scraped with 500 μL of sodium acetate 25 mM, pH 5.0, containing protease inhibitors (Life Technologies®, Carlsbad, CA, USA). Afterwards, 100 μL of sodium acetate buffer (blank) and each tissue extract was added, in triplicate, to the wells and incubated for 12 hours, at 37°C. After this step, the plates were washed three times with washing buffer (50 mM Tris-HCl, 150 mM NaCl, 0.05% Tween 20, 0.02 mM EDTA, 7.7 mM sodium azide, pH 7.75) and the remaining biotinylated HS was developed with europium-labeled streptavidin. Finally, 200 μL of enhancement solution (Perkin-Elmer, Wallac Oy, Turku, Finland) was added to release the europium bound to streptavidin, and determination of retained fluorescence was measured in a Victor3 time-resolved fluorometer (Perkin-Elmer, Wallac Oy, Turku, Finland). The product obtained by heparanase activity was determined by the ratio between the relative fluorescence intensity from tissue samples and the fluorescence of non-degraded heparan sulfate (blank). The relative activity of heparanase was then estimated using the fluorescence value obtained above and adjusted for the weight of each tissue sample (μg). Furthermore, it is important to point out that, for example, a higher fluorescence ratio represents low heparan sulfate degradation heparanase activity.

### Western blot

An assay was performed to verify the expression of different isoforms of HPSE2 (HPSE2a, HPSE2b and HPSE2c), the active (50 kDa) and precursor (65 kDa) forms of HPSE and also the expression of heparan sulfate (HS) using human thyroid samples (a normal thyroid specimen collected from a contra-lateral lobe of a patient with follicular adenoma submitted to total thyroidectomy and a fragment of papillary thyroid carcinoma pT2N0M0). Human tissue fragments were macerated using liquid nitrogen, and diluted in 150 μL of lysis buffer (50 mmol/L Tris–HCl, pH 7.4, 1% Tween 20, 0.25% sodium deoxycholate, 150 mmol/L NaCl, 1 mmol/L EGTA, 1 mmol/L Na3SO4, 1 mmol/L NaF) containing a protease inhibitor cocktail diluted to 1:100 (P8340, Sigma-Aldrich®, Saint Louis, MO, USA). The tissues were homogenized by sonication (3 cycles of 10 seconds, at 60 W, Unique®, São Paulo, Brazil). Protein extracts were cleared by centrifugation and protein concentration was determined using the Coomassie Blue G-250 protein assay. An equal volume of sodium dodecyl sulfate (SDS) gel loading buffer (100 mmol/L Tris–HCl, pH 6.8), 200 mmol/L dithiothreitol, 4% SDS, 0.1% bromophenol blue and 20% glycerol was added to the samples, which were subsequently boiled for 10 minutes. Equal quantities of total protein extract (50 μg) were loaded onto 15% SDS-PAGE and blotted onto nitrocellulose membranes (Millipore®, Billerica, MA, USA). Membranes were blocked using 2% BSA in Tris-buffered saline (TBS), washed with 0.05% Tween 20 (TBST buffer) and incubated overnight at 4°C with appropriate primary antibody anti-HPSE2 C-17 (this antibody recognizes the three isoformas of HPSE2: 2a, 2b and 2c), anti-HPSE H-80 (both at 1:1,000 dilution), anti-actin (I-19), at 1:3,000 dilution (Santa Cruz®, Santa Cruz, CA, USA) or anti-HS (biotin-conjugated anti-HS mouse IgG code 370262, Seikagaku Corporation®, Tokyo, Japan) at 1:100 dilution. After washing in TBST buffer, membranes were incubated with anti-rabbit or anti-goat HRP-conjugated secondary antibodies (Santa Cruz®, Santa Cruz, CA, USA), at 1:2,500 dilutions in blocking buffer for 1 h. Detection was performed using enhanced chemiluminescence (SuperSignal West Pico®, ThermoFisher Scientific®, Waltham, MA, USA). Western Blot images were obtained using DNR-MF-ChemiBis^®^ equipment (Uniscience^®^, Sao Paulo, Brazil) and quantified by densitometry using Scion Image Software® version 4.03 (Scion® Corporation, Frederick, Maryland, USA).

### Statistical analysis

The distributions were defined as non-parametric by the Kolmogorov-Smirnov test. For a descriptive analysis, qualitative data were expressed as absolute numbers and relative rates, and quantitative data as means and standard errors (SE). The Fisher exact test and Mann-Whitney test were used to compare phenomena between qualitative variables and between groups and quantitative variables, respectively. To test for accuracy, ROC (Receiver Operator Characteristic) analysis was employed and specificity, sensibility, predictive values and 95% confidence interval (95%CI) were calculated. For all analysis the probability of a making an α or type I error was equal to or less than 5% (P≤ 0.05) and SPSS^®^ version 17.0 (SPSS^®^ Inc; Illinois, USA) was used.

## Results

### Prospective study

**Fig 1:**
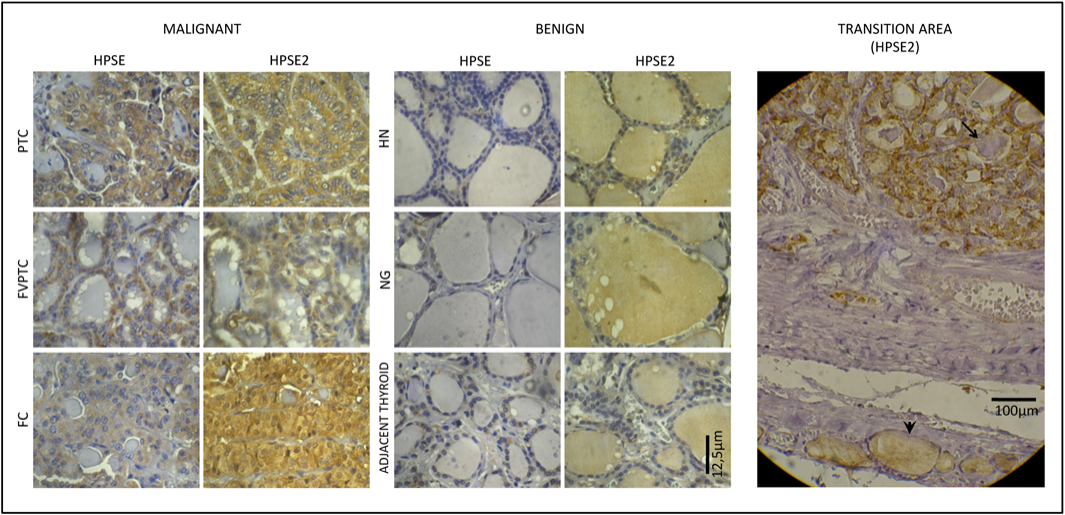
Immunostaining for HPSE and HPSE2 in malignant and benign thyroid samples. Left panel represents malignant thyroid tissues (PTC, papillary thyroid carcinoma; FVPTC, follicular variant of papillary thyroid carcinoma and FC, follicular carcinoma), whilst the middle panel represents benign thyroid tissues (HN, hyperplasic nodule, NG nodular goiter and normal adjacent thyroid tissue). Immunohistochemistry obtained using optical microscopy, X400. Right Panel (Transition area) demonstrates different pattern of HPSE2 labeling, showing adjacent normal thyroid tissue (arrow head) and follicular carcinoma (arrow) in the same photomicrography. Immunohistochemistry was obtained using light microscopy, X200. Legend: * represent colloidal areas in each photomicrography.

To investigate the protein expression and differential protein profile among malignant, benign and normal thyroid lesions, we performed immunohistochemistry analyses for HPSE and HPSE2 on paraffin-embedded section. As shown in Fig 1, HPSE staining was clearly positive in follicular cells and negative in colloid for all 15 cases of DTC and in 10 cases of benign lesions (83.3%) but in a weaker labeling. A similar staining pattern was obtained for normal adjacent thyroid areas in comparison with benign lesions, but also with a weaker labeling. Interestingly, HPSE2 immunostaining showed a markedly higher expression in malignant tissue if compared with counterpart benign tumors. Moreover, high expression of HPSE2 was noted in almost half (41.7%) of benign thyroid colloid areas whilst no immunostaining was found in colloidal areas for all 15 DTC cases. When the tissues that present normal adjacent thyroid areas were analyzed, none of them (22 samples: 100%) presented staining of HPSE2 only in colloid and not in follicular cells.

**Fig 2:**
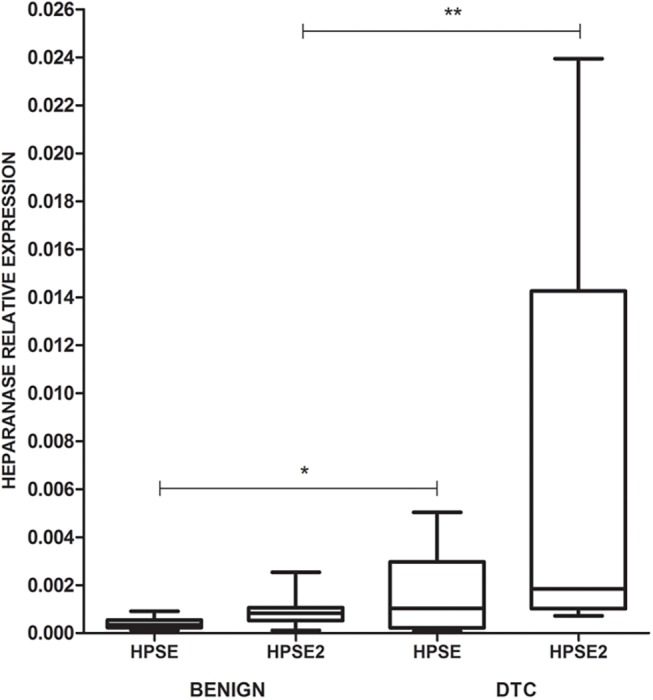
HPSE and HPSE2 quantitative RT-PCR analysis. The values are represented by box plots and demonstrate the high expression of HPSE2 in DTC (**P = 0.001, Mann-Whitney test) and also of HPSE but with just a statistical trend (*P = 0.067, Mann-Whitney test); (N = 27).

Then, we performed real time RT-PCR to confirm that there is increased expression of both heparanases in thyroid carcinomas compared to benign tumors. Fig 2 shows that there was around seven times more relative HPSE2 expression in DTC compared to the benign samples, respectively, (62.9 ± 25.0) x 10^−4^ and (9.1 ± 1.7) x 10^−4^ (P = 0.001, Mann-Whitney test). A similar result was obtained for HPSE analysis with four times more expression in DTC (15.4 ± 4.6) x 10^−4^ compared to benign lesions (4.4 ± 0.6) x 10^−4^ although this difference was just a statistically trend (P = 0.067, Mann-Whitney test).

Proteolytic cleavage from pre-HPSE (65 kDa) to obtain active enzyme (50 kDa) occurs between glutamic acid^109^ and lysine^158^ residues. Since HPA1 (H-80) antibody is a rabbit polyclonal antibody raised against amino acids 101–180 mapping within an internal region of human HPSE, this specific antibody is able to identify both HPSE isoforms in the immunoassay. Further, RT-PCR analysis amplifies both HPSE isoforms (50 and 65 kDa), due to the fact that the forward and reverse primers identify positions 1142–1165 and 1194–1216, respectively located in the C-terminus of heparanase cDNA.

Given that HPA1 antibody is able to identify both HPSE isoforms in the immunohistochemistry, and RT-PCR analysis also amplifies pre- and active enzyme, we performed an enzymatic assay to verify the real action of this enzyme in normal and tumoral tissues. As shown in Table 1, a significant increase of HPSE activity was observed in DTC (4.8 ± 0.1 relative fluorescence/μg tissue) compared to the benign thyroid lesions (8.5 ± 0.4 relative fluorescence/μg tissue) (P<0.0001, Mann-Whitney test).

**Table 1:** Heparanase enzymatic assay in DTC and benign thyroid lesions (N = 27).

	Activity (relative fluorescence** / μg tissue)	Group (relative fluorescencee** / μg tissue)
**Differentiated Thyroid Carcinoma**		
Follicular Variant of PTC	4.9	
Papillary Thyroid Carcinoma	5.2	4.8 ± 0.1***
Follicular Carcinoma	4.3	
**Benign Lesions**		
Follicular Adenoma	8.4	
Hyperplastic Nodule	9.3	8.5 ± 0.4***
Hashimoto Thyroiditis	7.7	
**P-Value** *	-	**<0.0001**

* Mann-Whitney test.

** Relative fluorescence expresses the ratio between intensity of fluorescence from degraded heparan sulfate and non-degraded substrate.

*** Mean ± standard error

Legend: PTC, papillary thyroid carcinoma.

**Fig 3:**
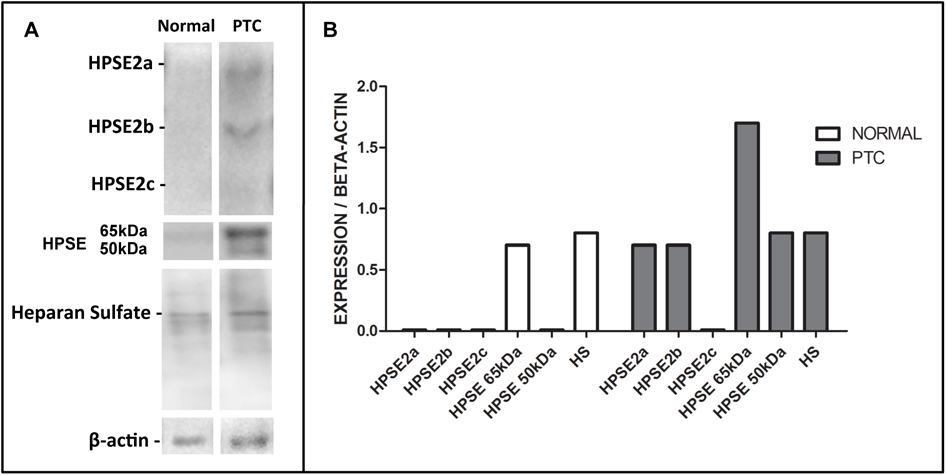
Proteins and heparan sulfate expression in thyroid tissues. (A), Expression of heparanase-2 isoforms (HPSE2a, HPSE2b and HPSE2c); heparanase active enzyme (50 kDa) and pro-enzyme (65 kDa); heparan sulfate (HS). The samples were analyzed on a single sample of normal thyroid sample (“Normal”), and papillary thyroid carcinoma (“PTC”). (B), The values indicate relative expression obtained by the ratio of proteins and HS corrected by beta-actin expression.

Finally, to validate how the isoforms of heparanases were expressed in normal thyroid tissue and also in DTC, an exploratory Western blot assay was performed (Fig 3). Surgically resected tissue samples were obtained from a normal thyroid specimen and a fragment of papillary thyroid carcinoma (PTC) as described in methods. As shown in Fig 2, virtually only the pro-enzyme (65 kDa) was detect in the normal thyroid sample. On the other hand, DTC showed a higher amount of both active and inactive HPSE isoform as well as higher HPSE2a and HPSE2b content.

**Fig 4:**
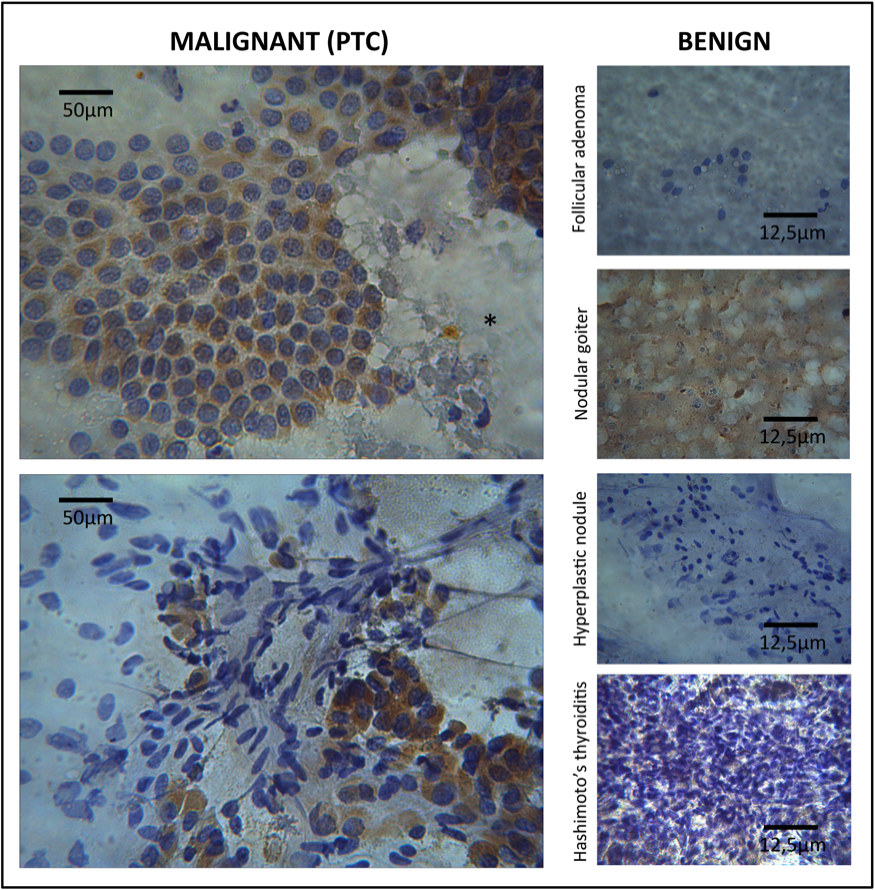
Immunostaining for HPSE2 in malignant and benign FNAB samples. Left panel represents malignant thyroid tissues (two cases of PTC) and the right panel represents benign thyroid tissues. Immunocytochemistry obtained using light microscopy, X400, showed a different pattern of HPSE2 staining: high expression on follicular cells of PTC and none expression on benign lesions and also a negative colloid expression on PTC (*) and positive on nodular goiter.

Trying to establish a preoperative diagnostic role of HPSE2, a preliminary assay with FNAB specimens was conduced. Six cases (2 PTC, and 4 benign lesions) were submitted to immunocytochemistry and a similar pattern was also obtained as demonstrated in Fig 4. Both cases of PTC presented high cytoplasmatic expression of HPSE2 on follicular cells and absence in colloidal areas when present; benign lesions demonstrated no expression of HPSE2 with just one case of colloidal expression in a nodular goiter.

### Retrospective study

Once a well distinguished immunohistochemical distribution of heparanases was established in the different compartments of the thyroid follicle, especially the presence of HPSE2 in DTC cells, immunohistochemistry for HPSE2 was evaluated as a possible diagnostic test to distinguish DTC from benign lesions in 230 selected samples as previously reported.

HPSE2 expression was detected in the neoplastic cells of all 61 specimens of DTC (including classic and follicular variant of PTC and also in all cases of follicular carcinomas) and in 79 cases (46.7%) of benign lesions. Interestingly, colloidal staining of HPSE2 was negative in all cases of DTC and positive in 111 benign samples (66.7%). When all tissues (221 samples) from non-neoplastic adjacent thyroid were analyzed, the same pattern of HPSE2 immunostaining was noted, positive in colloid and negative in follicular cells. In light of these results, a relationship was established between HPSE2 expression and diagnosis of thyroid lesions (benign or malignant): HPSE2 expression was positive in neoplastic cells of DTC and negative on colloid, while in benign lesions HPSE2 was observed preferentially in colloidal areas (P<0.0001, Fisher’s exact test).

When the association of positive HPSE2 staining in neoplastic cells and negative in colloid was compared to all other possible associations (Table 2), it was clearly noted that this HPSE2 pattern could be used as a possible diagnostic test for DTC (P<0.0001, Fisher’s exact test).

**Table 2:** HPSE2 patterns of immunostaining in DTC and benign lesions.

	HPSE2 immunostaining	
	Positive in cells and negative in colloid	Other associations*
**DTC**	61 (100%)	0 (0%)
**Benign Lesions**	47 (27.8%)	122 (72.2%)

The values represent the number of cases and percentage (%); P<0.0001 (Fisher’s exact test)

* other associations represent heparanase negative in cells and positive in colloid, positive in both or negative in both.

Legend: DTC, differentiated thyroid carcinoma; HPSE2, Heparanase 2

Finally, the validation of positive HPSE2 staining in follicular cells and negative in colloid as a diagnostic test for DTC was tested. This pattern of HPSE2 localization proved to have a sensitivity of 100% (CI95%: 94.1%-100.0%), specificity of 72.2% (CI95%: 64.8%-78.8%), positive predictive value of 56.5% (CI95%: 46.6%-66.6%), negative predictive value of 100% (CI95%: 97.0%-100.0%) and high accuracy with an area under the ROC curve of 86.1% (CI95%: 80.9%-90.3%). Of the 230 cases analyzed, 61 true-positive tests and no false-negative cases were found. On the other hand, there were 122 true-negative tests and 47 (27.8%) cases of false-positive results (18.3% for FA, 36.7% for HN and 30.0% for NG).

## Discussion

The present study demonstrated that both heparanases (HPSE and HPSE2) possibly play an important role in thyroid carcinogenesis and might also be used as a differential diagnosis for benign and malignant follicular tumors. HPSE2 immunohistochemistry is a diagnostic test with high accuracy, without false-negative results. It was also noted that enhanced HPSE2 expression and high levels of HPSE enzymatic activity may be strongly indicative of DTC, suggesting that both heparanases may be directly involved in the carcinogenesis of differentiated thyroid carcinoma. Based on our results and also on well established on specific literature, we propose a hypothetical model for best explain the role of heparanases in thyroid carcinogenesis.

**Fig 5:**
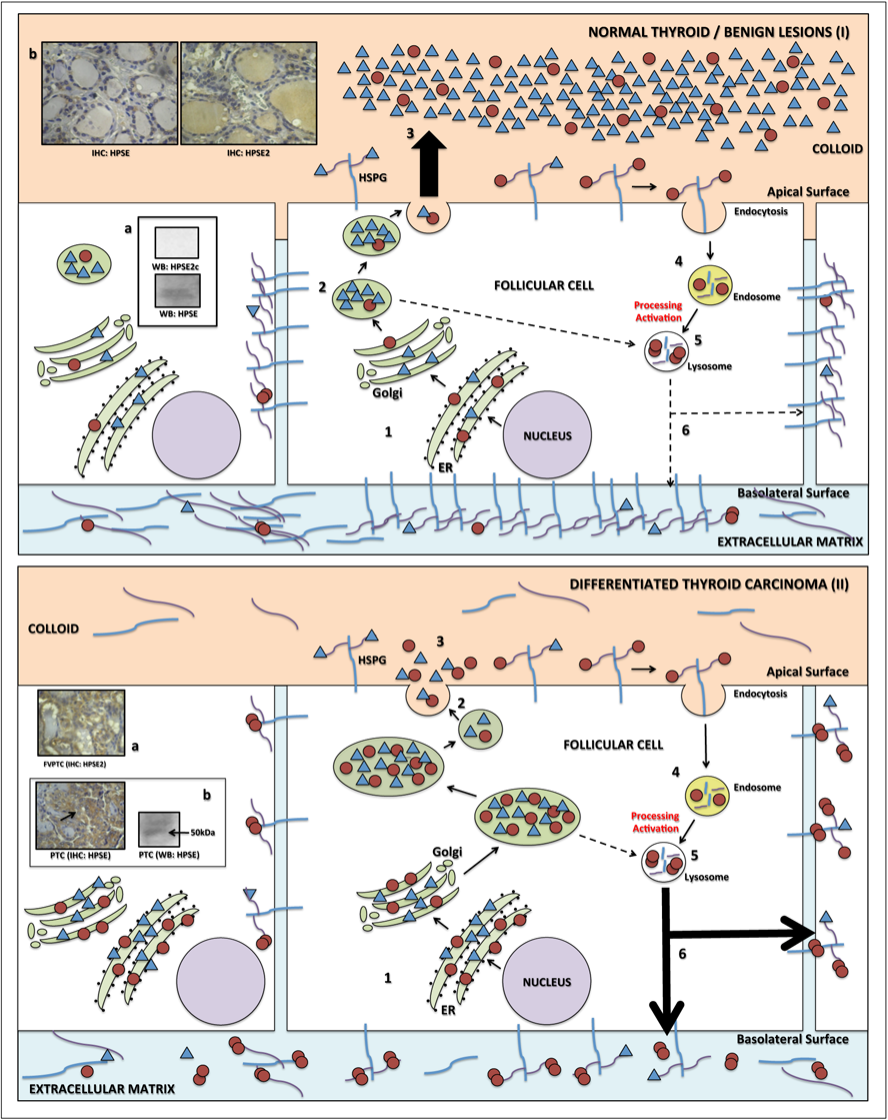
Hypothetical scheme to explain herapanase physiology based on data of this study and also from Gengis-Velitski et al. [24], Levy-Adam et al. 2010 [25], Linke et al. 2002 [26], Zetzer et al. 2004 [27] and Nadav et al 2002 [17]. (I) In the normal thyroid follicle or benign lesions HPSE and HPSE2 are first targeted to the endoplasmic reticulum lumen (ER), shuttled to the Golgi apparatus (*step 1*), partially processed and activated in lysosomes (*step 2*), and both heparanases (HPSE and HPSE2) are highly secreted to colloid (*step 3*); HPSE is therefore partially internalized (*step 4*) and also activated in the lysosomes (*step 5*) and directed to the basolateral surface of follicular thyroid cells where it presents low activity (*step 6*). (II) In the differentiated thyroid carcinoma there is overexpression of both heparanases (*step 1*), increased processing and activation of HPSE in lysosomes (*step 2*) and low secretion to colloid (*step 3*) and follows that described in steps 4 and 5 below. An increased secretion of HPSE to basolateral surface and extracellular matrix occurs to degrade HSPG (*step 6*, large arrows). * Additional information: (Ia) low presence of latent HPSE and no presence of HPSE2 in the cytoplasm of normal / benign lesion follicular cell (Western blotting); (Ib) immunoexpression of both heparanases by immunohistochemistry: low positivity in follicular cells for HPSE and positive staining in colloid for HPSE2; (IIa) high immunostaining in neoplastic cells for HPSE2; (IIb) high immunostaining in neoplastic cells for HPSE and Western blotting demonstrating the presence of the active form (50 kDa). Legend: HPSE 65 kDa, ο; HPSE 50 kDa, οο; HPSE2, Δ.

As previously reported, both heparanases are first targeted to the endoplasmic reticulum lumen (through a specific signal peptide), and then shuttled to the Golgi apparatus to be subsequently secreted. [24] The immunohistochemistry profile for HPSE and HPSE2 in the normal thyroid follicle and also in benign lesions (Fig 5-I) suggest that both HPSE and HPSE2 are secreted to colloid in order to degrade and process HS proteoglycans [25] and, probably, could play a role in a normal thyroglobulin processing [26]. In other words, HPSE would be partially activated [27], as confirmed by the virtual absence of the 50 kDa HPSE isoform in normal thyroid sample and, thus, both HPSE and especially HPSE2 would remain preferentially in colloid in the benign tumors and normal thyroid tissues.

On the other hand, in DTC follicular cells (Fig 5-II) there is overexpression of both heparanases (as demonstrated by immunohistochemistry and qRT-PCR). This process is followed by HPSE translocation through the Golgi apparatus and trans-Golgi network to endosomes/lysosomes, where this enzyme is mostly processed and activated (50 kDa). Therefore, active HPSE should participate in degradation of HSPG from the basolateral surface, basement membrane and extracellular matrix of malignant lesions [24], which directly promotes loss of cell adherence promoting increased cellular migration and consequent tumor invasion. A small amount of heparanase is still secreted to colloid and rapidly interacts with cell membrane HSPG: the complexes HPSE2-HSPG and HPSE-HSPG should, then, undergo endocytosis followed by intracellular proteolysis, as proposed by Nadav and colleagues. [17]

As previously mentioned, there are not many studies concerning heparanases and thyroid tumors [13, 14, 28]. The most relevant data demonstrated an overexpression of HPSE in papillary thyroid carcinoma compared to follicular adenomas and thyroid neoplastic cell lines [14]. HPSE overexpression correlates with the invasive potential of the tumor *in vitro*. The immunofluorescence analysis of HSPG conducted in the same study revealed that heparan sulfate was abundantly deposited in the basement membrane of normal thyroid follicles and in benign follicular adenomas, but was absent in most thyroid papillary carcinomas, suggesting that HPSE has a functional role in the extracellular matrix. It is important to point out that the author had just studied the role of HPSE and HS on the extracellular matrix, but did not attempt to evaluate such molecules in colloid. This study also showed that in normal thyroid and also in follicular adenoma HS is present in the basement membrane, whilst it is absent in colloid. However, in PTC the opposite pattern was observed, where HS is absent in basement membrane and is highly present in colloid. The authors suggest that the loss of HS content in the basement membrane is caused, in part, by the HPSE-mediated HS degradation, demonstrating an inverse correlation between HS composition and HPSE expression [29]. Furthermore, some secreted HSGPs, such as syndecan-1 and glypican-1, are increased in a variety of malignancies such as thyroid [29], pancreatic cancer [30, 31], breast cancer and myeloma [32]. Therefore, as proposed by other authors [14, 33–36], malignant cells probably have high HSPG expression on the apical surface, especially syndecan family members, and increased secretion of HS to colloid.

Certainly one of the most important results of the present work was to demonstrate that HPSE2 might be a potential immunomarker for the differential diagnosis of DTC. Many studies have demonstrated the overexpression of heparanase and HPSE2, especially in invasive solid tumors in comparison with benign lesions.[37] However, there is little data demonstrating that these proteins are valid markers for malignant tumor diagnosis. High rates of heparanase in circulating lymphocytes have been associated with recurrence in breast cancer patients[38], and a decrease in plasmatic levels has also been associated with therapeutic response in children with Hodgkin lymphoma[39]. Zhang and colleagues identified heparanase as good marker for ovarian cancer metastasis with sensitivity of 69.6% and specificity of 67.2%[40]. Likewise, HPSE2 was suggested as a good tumor marker, better than HPSE, for the first time only in 2008 in colorectal carcinoma in comparison with normal mucosa with sensitivity of 98% and specificity of 100% using immunohistochemistry technique[18]. Moreover, HPSE2 was also associated with the diagnosis of ovarian cancer with accuracy of 78%[41, 42] and recently with better outcomes in patients with gastric cancer[43].

In fact, the prospective study demonstrated that HPSE2 expression presents different localization patterns in benign and malignant lesions. Positive HPSE2 staining in neoplastic cells and negative staining in colloid suggest a diagnostic test for DTC with high accuracy and, more importantly, without false-negatives. It is important to point out that this result was obtained by applying a well-established, low-cost, broadcast immunohistochemistry assay.

Many studies employ immunohistochemistry techniques in an attempt to search for markers involved in the genesis or in specific characteristics of follicular patterned tumors. The most employed markers applied in the distinction between benign and malignant thyroid lesions are cytokeratin-19 (CK-19), galectin-3 (Gal-3) and the Hector Battifora mesothelial-1 (HBME-1). A diagnostic meta-analysis showed that the positivity of CK-19 for the diagnosis of malignant thyroid lesions demonstrated global sensitivity of 81% and specificity of 73%; for Gal-3, sensitivity of 82% and specificity of 81%; and for HBME-1, sensitivity of 77% and specificity of 83%. The association of the three markers determined sensitivity of 85%, specificity of 97%, and diagnostic odds ratio of 95.1, but false-result rates still persist [7]. This is an important reason to search for an accurate immunological marker. The present study showed that HPSE2, used as a single marker for DTC, achieved accuracy of 86.1%, as high as observed in the other three markers, without false-negative cases, which means this molecule has great potential as a marker in the differential diagnosis of thyroid nodule.

Other studies have described the development of gene expression to better distinguish thyroid lesions [44, 45]. A recently published article studied 413 cytologically indeterminate aspirates submitted to a microarray evaluation of 167 genes (Veracyte Inc®, San Francisco, CA, USA). When compared to the standard histopathological exam 92% sensitivity and high rates of negative predictive value were obtained, but with seven cases of false-negative results [45].

Another important result of the present study was the expression of HPSE2 in FNAB specimens. It was just a preliminary study but these findings are in accordance with the immunohistochemistry test and affirm once more the need of a specific study with a large sample size is necessary on this field. The authors suggest that for this validation is necessary a specific study with a larger sample and also with a specific methodology as proposed by Ferraz et al.[46]: in a review article including 20 other manuscripts demonstrated that the role of immunocytochemistry in the differentiation of follicular-pattern lesions needs methodological improvements and standardizations. One of the limitations of the present study concerns the use of tissue samples, which do not allow a preoperative evaluation that could possibly avoid unnecessary thyroidectomies. It is imperative that other prospective studies and double-blind evaluations be conducted to test the reproducibility of these cytological findings, such as specimens obtained by ultrasound-guided fine needle aspiration.

In conclusion, differential localization of HPSE2 in the thyroid follicle compared to DTC may suggest a role of heparanase in thyroid carcinogenesis. Also, HPSE2 was established as an accurate marker in the distinction between benign and malignant thyroid tumors with real potential application for diagnosis in clinical practice.

## Supporting Information

S1 Fig Specificity of the anti-heparanase-2 antibody. *Heparanase-2 antibody*, HPA2 C-17 (sc14900; Santa Cruz®, Biotechnology, CA, USA) was used at a final concentration of 2 μg/mL; *Heparanase-2 antibody + peptide*, Blocking assay combining the antibody HPA2 C-17 at 2 μg/mL with five-fold excess (10 μg/mL) of blocking peptide (sc14900P; Santa Cruz®, Biotechnology, CA, USA), incubated overnight at 4°C, as suggested by manufacturer's instructions, followed by immunohistochemistry assay described in methods. *Negative control*, immunohistochemistry assay was performed in the absence of primary antibody. There was no immunostaining in follicular cells of pappilary thyroid carcinoma and also in colloid of a hyperplasic nodule using heparanase-2 antibody. The images were obtained using light microscopy. The bars represent 105 μm.
(TIF)

## References

[1] Yassa L, Cibas ES, Benson CB, Frates MC, Doubilet PM, Gawande AA, et al Long-term assessment of a multidisciplinary approach to thyroid nodule diagnostic evaluation. Cancer. 2007;111(6):508–16. Epub 2007/11/14. 10.1002/cncr.23116 . 17999413

[2] Cooper DS, Doherty GM, Haugen BR, Kloos RT, Lee SL, Mandel SJ, et al Revised American Thyroid Association management guidelines for patients with thyroid nodules and differentiated thyroid cancer. Thyroid. 2009;19(11):1167–214. Epub 2009/10/29. 10.1089/thy.2009.0110 . 19860577

[3] Wang CC, Friedman L, Kennedy GC, Wang H, Kebebew E, Steward DL, et al A large multicenter correlation study of thyroid nodule cytopathology and histopathology. Thyroid. 2011;21(3):243–51. Epub 2010/12/31. 10.1089/thy.2010.0243 . 21190442 PMC3698689

[4] Hodak SP, Rosenthal DS. Information for clinicians: commercially available molecular diagnosis testing in the evaluation of thyroid nodule fine-needle aspiration specimens. Thyroid. 2013;23(2):131–4. Epub 2012/09/19. 10.1089/thy.2012.0320 . 22984796

[5] Brown LM, Helmke SM, Hunsucker SW, Netea-Maier RT, Chiang SA, Heinz DE, et al Quantitative and qualitative differences in protein expression between papillary thyroid carcinoma and normal thyroid tissue. Molecular carcinogenesis. 2006;45(8):613–26. 10.1002/mc.20193 16788983 PMC1899163

[6] Myers MB, McKim KL, Parsons BL. A subset of papillary thyroid carcinomas contain KRAS mutant subpopulations at levels above normal thyroid. Molecular carcinogenesis. 2014;53(2):159–67. 10.1002/mc.21953 . 22930660

[7] Matos LL, Del Giglio AB, Matsubayashi CO, Farah ML, Del Giglio A, Pinhal MA. Expression of ck-19, galectin-3 and hbme-1 in the differentiation of thyroid lesions: systematic review and diagnostic meta-analysis. Diagn Pathol. 2012;7(1):97. Epub 2012/08/15. doi: 1746-1596-7-97 [pii] 10.1186/1746-1596-7-97 . 22888980 PMC3523001

[8] Saggiorato E, De Pompa R, Volante M, Cappia S, Arecco F, Dei Tos AP, et al Characterization of thyroid 'follicular neoplasms' in fine-needle aspiration cytological specimens using a panel of immunohistochemical markers: a proposal for clinical application. Endocr Relat Cancer. 2005;12(2):305–17. Epub 2005/06/11. doi: 12/2/305 [pii] 10.1677/erc.1.00944 . 15947105

[9] McAllister SS, Weinberg RA. Tumor-host interactions: a far-reaching relationship. J Clin Oncol. 2010;28(26):4022–8. Epub 2010/07/21. 10.1200/JCO.2010.28.4257 JCO.2010.28.4257 [pii]. . 20644094

[10] Ilan N, Elkin M, Vlodavsky I. Regulation, function and clinical significance of heparanase in cancer metastasis and angiogenesis. Int J Biochem Cell Biol. 2006;38(12):2018–39. Epub 2006/08/12. doi: S1357-2725(06)00191-9 [pii] 10.1016/j.biocel.2006.06.004 . 16901744

[11] Vlodavsky I, Elkin M, Abboud-Jarrous G, Levi-Adam F, Fuks L, Shafat I, et al Heparanase: one molecule with multiple functions in cancer progression. Connect Tissue Res. 2008;49(3):207–10. Epub 2008/07/29. doi: 795328198 [pii] 10.1080/03008200802143281 . 18661344

[12] Chen L, Sanderson RD. Heparanase regulates levels of syndecan-1 in the nucleus. PLoS One. 2009;4(3):e4947 Epub 2009/03/24. 10.1371/journal.pone.0004947 19305494 PMC2654539

[13] Buchs AE, Zehavi S, Sher O, Yeheskely E, Muggia-Sulam M, Sherman Y, et al Heparanase, galectin-3, and tissue factor mRNA are expressed in benign neoplasms of the thyroid. Endocrine. 2003;22(2):81–4. Epub 2003/12/11. doi: ENDO:22:2:81 [pii] 10.1385/ENDO:22:2:81 . 14665710

[14] Xu X, Quiros RM, Maxhimer JB, Jiang P, Marcinek R, Ain KB, et al Inverse correlation between heparan sulfate composition and heparanase-1 gene expression in thyroid papillary carcinomas: a potential role in tumor metastasis. Clin Cancer Res. 2003;9(16 Pt 1):5968–79. Epub 2003/12/17. . 14676122

[15] McKenzie E, Tyson K, Stamps A, Smith P, Turner P, Barry R, et al Cloning and expression profiling of Hpa2, a novel mammalian heparanase family member. Biochem Biophys Res Commun. 2000;276(3):1170–7. Epub 2000/10/12. 10.1006/bbrc.2000.3586 S0006-291X(00)93586-1 [pii]. . 11027606

[16] Dempsey LA, Brunn GJ, Platt JL. Heparanase, a potential regulator of cell-matrix interactions. Trends Biochem Sci. 2000;25(8):349–51. Epub 2000/08/01. doi: S0968-0004(00)01619-4 [pii]. . 10916150 10.1016/s0968-0004(00)01619-4

[17] Nadav L, Eldor A, Yacoby-Zeevi O, Zamir E, Pecker I, Ilan N, et al Activation, processing and trafficking of extracellular heparanase by primary human fibroblasts. Journal of cell science. 2002;115(Pt 10):2179–87. Epub 2002/04/26. . 11973358 10.1242/jcs.115.10.2179

[18] Peretti T, Waisberg J, Mader AM, de Matos LL, da Costa RB, Conceicao GM, et al Heparanase-2, syndecan-1, and extracellular matrix remodeling in colorectal carcinoma. European journal of gastroenterology & hepatology. 2008;20(8):756–65. 10.1097/MEG.0b013e3282fc2649 . 18617780

[19] Suarez ER, Paredes-Gamero EJ, Del Giglio A, Tersariol IL, Nader HB, da Pinhal MA. Heparan sulfate mediates trastuzumab effect in breast cancer cells. BMC Cancer. 2013;13(1):444. Epub 2013/10/03. doi: 1471-2407-13-444 [pii] 10.1186/1471-2407-13-444 . 24083474 PMC3850728

[20] Rodrigues LM, Oliveira LZ, Pinhal MA. Expression of heparanase isoforms in intervertebral discs classified according to Pfirrmann grading system for disc degeneration. Spine (Phila Pa 1976). 2013;38(13):1112–8. Epub 2013/02/02. 10.1097/BRS.0b013e3182894cf4 . 23370684

[21] Behzad F, Brenchley PE. A multiwell format assay for heparanase. Anal Biochem. 2003;320(2):207–13. Epub 2003/08/21. doi: S0003269703003580 [pii]. . 12927826 10.1016/s0003-2697(03)00358-0

[22] Boucas RI, Trindade ES, Tersariol IL, Dietrich CP, Nader HB. Development of an enzyme-linked immunosorbent assay (ELISA)-like fluorescence assay to investigate the interactions of glycosaminoglycans to cells. Anal Chim Acta. 2008;618(2):218–26. Epub 2008/06/03. doi: S0003-2670(08)00808-8 [pii] 10.1016/j.aca.2008.04.059 . 18513543

[23] Trindade ES, Oliver C, Jamur MC, Rocha HA, Franco CR, Boucas RI, et al The binding of heparin to the extracellular matrix of endothelial cells up-regulates the synthesis of an antithrombotic heparan sulfate proteoglycan. J Cell Physiol. 2008;217(2):328–37. Epub 2008/06/11. 10.1002/jcp.21504 . 18543288

[24] Gingis-Velitski S, Zetser A, Kaplan V, Ben-Zaken O, Cohen E, Levy-Adam F, et al Heparanase uptake is mediated by cell membrane heparan sulfate proteoglycans. J Biol Chem. 2004;279(42):44084–92. Epub 2004/08/05. 10.1074/jbc.M402131200 M402131200 [pii]. . 15292202

[25] Levy-Adam F, Feld S, Cohen-Kaplan V, Shteingauz A, Gross M, Arvatz G, et al Heparanase 2 interacts with heparan sulfate with high affinity and inhibits heparanase activity. J Biol Chem. 2010;285(36):28010–9. Epub 2010/06/26. doi: M110.116384 [pii] 10.1074/jbc.M110.116384 20576607 PMC2934666

[26] Linke M, Herzog V, Brix K. Trafficking of lysosomal cathepsin B-green fluorescent protein to the surface of thyroid epithelial cells involves the endosomal/lysosomal compartment. Journal of cell science. 2002;115(Pt 24):4877–89. . 12432075 10.1242/jcs.00184

[27] Zetser A, Levy-Adam F, Kaplan V, Gingis-Velitski S, Bashenko Y, Schubert S, et al Processing and activation of latent heparanase occurs in lysosomes. Journal of cell science. 2004;117(Pt 11):2249–58. Epub 2004/05/06. 10.1242/jcs.01068 117/11/2249 [pii]. . 15126626

[28] Jiang P, Kumar A, Parrillo JE, Dempsey LA, Platt JL, Prinz RA, et al Cloning and characterization of the human heparanase-1 (HPR1) gene promoter: role of GA-binding protein and Sp1 in regulating HPR1 basal promoter activity. J Biol Chem. 2002;277(11):8989–98. Epub 2002/01/10. 10.1074/jbc.M105682200 M105682200 [pii]. . 11779847

[29] Lu S, Huang M, Kobayashi Y, Komiyama A, Li X, Katoh R, et al Alterations of basement membrane in di-isopropanolnitrosamine-induced carcinogenesis of the rat thyroid gland: an immunohistochemical study. Virchows Arch. 2000;436(6):595–601. Epub 2000/08/05. . 10917175 10.1007/s004280000180

[30] Conejo JR, Kleeff J, Koliopanos A, Matsuda K, Zhu ZW, Goecke H, et al Syndecan-1 expression is up-regulated in pancreatic but not in other gastrointestinal cancers. Int J Cancer. 2000;88(1):12–20. Epub 2000/08/30. 10.1002/1097-0215(20001001)88:1<12::AID-IJC3>3.0.CO;2-T [pii]. . 10962434

[31] Kleeff J, Ishiwata T, Kumbasar A, Friess H, Buchler MW, Lander AD, et al The cell-surface heparan sulfate proteoglycan glypican-1 regulates growth factor action in pancreatic carcinoma cells and is overexpressed in human pancreatic cancer. J Clin Invest. 1998;102(9):1662–73. Epub 1998/11/05. 10.1172/JCI4105 9802880 PMC509114

[32] Thompson CA, Purushothaman A, Ramani VC, Vlodavsky I, Sanderson RD. Heparanase regulates secretion, composition, and function of tumor cell-derived exosomes. J Biol Chem. 2013;288(14):10093–9. Epub 2013/02/23. 10.1074/jbc.C112.444562 C112.444562 [pii]. 23430739 PMC3617250

[33] Bologna-Molina R, Gonzalez-Gonzalez R, Mosqueda-Taylor A, Molina-Frechero N, Damian-Matsumura P, Dominguez-Malagon H. Expression of syndecan-1 in papillary carcinoma of the thyroid with extracapsular invasion. Arch Med Res. 2010;41(1):33–7. Epub 2010/05/01. doi: S0188-4409(09)00211-2 [pii] 10.1016/j.arcmed.2009.11.004 . 20430252

[34] Yamanaka K, Ito Y, Okuyama N, Noda K, Matsumoto H, Yoshida H, et al Immunohistochemical study of glypican 3 in thyroid cancer. Oncology. 2007;73(5–6):389–94. Epub 2008/05/31. doi: 000136159 [pii] 10.1159/000136159 . 18511877

[35] Mitselou A, Ioachim E, Peschos D, Charalabopoulos K, Michael M, Agnantis NJ, et al E-cadherin adhesion molecule and syndecan-1 expression in various thyroid pathologies. Exp Oncol. 2007;29(1):54–60. Epub 2007/04/14. doi: 30/599 [pii]. . 17431390

[36] Ito Y, Yoshida H, Nakano K, Takamura Y, Miya A, Kobayashi K, et al Syndecan-1 expression in thyroid carcinoma: stromal expression followed by epithelial expression is significantly correlated with dedifferentiation. Histopathology. 2003;43(2):157–64. Epub 2003/07/25. doi: 1656 [pii]. . 12877731 10.1046/j.1365-2559.2003.01656.x

[37] Arvatz G, Shafat I, Levy-Adam F, Ilan N, Vlodavsky I. The heparanase system and tumor metastasis: is heparanase the seed and soil? Cancer Metastasis Rev. 2011;30(2):253–68. Epub 2011/02/11. 10.1007/s10555-011-9288-x . 21308479

[38] Theodoro TR, de Matos LL, Sant Anna AV, Fonseca FL, Semedo P, Martins LC, et al Heparanase expression in circulating lymphocytes of breast cancer patients depends on the presence of the primary tumor and/or systemic metastasis. Neoplasia. 2007;9(6):504–10. Epub 2007/07/03. 17603633 10.1593/neo.07241 PMC1899258

[39] Ben Arush MW, Shafat I, Ben Barak A, Shalom RB, Vlodavsky I, Ilan N. Plasma heparanase as a significant marker of treatment response in children with Hodgkin lymphoma: pilot study. Pediatr Hematol Oncol. 2009;26(4):157–64. Epub 2009/05/14. doi: 911106651 [pii] 10.1080/08880010902754917 . 19437318

[40] Zhang W, Yang HC, Wang Q, Yang ZJ, Chen H, Wang SM, et al Clinical value of combined detection of serum matrix metalloproteinase-9, heparanase, and cathepsin for determining ovarian cancer invasion and metastasis. Anticancer Res. 2011;31(10):3423–8. Epub 2011/10/04. doi: 31/10/3423 [pii]. . 21965756

[41] Marques RM, Focchi GR, Theodoro TR, Castelo A, Pinhal MA, Nicolau SM. The immunoexpression of heparanase 2 in normal epithelium, intraepithelial, and invasive squamous neoplasia of the cervix. J Low Genit Tract Dis. 2012;16(3):256–62. Epub 2012/03/29. 10.1097/LGT.0b013e3182422c69 . 22453758

[42] de Moura JP Jr, Nicolau SM, Stavale JN, da Silva Pinhal MA, de Matos LL, Baracat EC, et al Heparanase-2 expression in normal ovarian epithelium and in benign and malignant ovarian tumors. Int J Gynecol Cancer. 2009;19(9):1494–500. Epub 2009/12/04. 10.1111/IGC.0b013e3181a834a2 00009577-200912000-00006 [pii]. . 19955924

[43] Zhang X, Xu S, Tan Q, Liu L. High expression of heparanase-2 is an independent prognostic parameter for favorable survival in gastric cancer patients. Cancer Epidemiol. 2013. Epub 2013/10/22. doi: S1877-7821(13)00154-9 [pii] 10.1016/j.canep.2013.09.012 . 24139593

[44] Eszlinger M, Paschke R. Molecular fine-needle aspiration biopsy diagnosis of thyroid nodules by tumor specific mutations and gene expression patterns. Mol Cell Endocrinol. 2010;322(1–2):29–37. Epub 2010/01/20. doi: S0303-7207(10)00012-2 [pii] 10.1016/j.mce.2010.01.010 . 20083161

[45] Alexander EK, Kennedy GC, Baloch ZW, Cibas ES, Chudova D, Diggans J, et al Preoperative Diagnosis of Benign Thyroid Nodules with Indeterminate Cytology. N Engl J Med. 2012 Epub 2012/06/27. 10.1056/NEJMoa1203208 . 22731672

[46] Ferraz C, Eszlinger M, Paschke R. Current state and future perspective of molecular diagnosis of fine-needle aspiration biopsy of thyroid nodules. The Journal of clinical endocrinology and metabolism. 2011;96(7):2016–26. Epub 2011/05/20. 10.1210/jc.2010-2567 . 21593119

