# Supplementary figures and images for: The Profile of Heparanase Expression Distinguishes Differentiated Thyroid Carcinoma from Benign Neoplasms
Source: PLoS One. 2015 Oct 21;10(10):e0141139. doi: 10.1371/journal.pone.0141139 (PMC4619411; doi:10.1371/journal.pone.0141139)

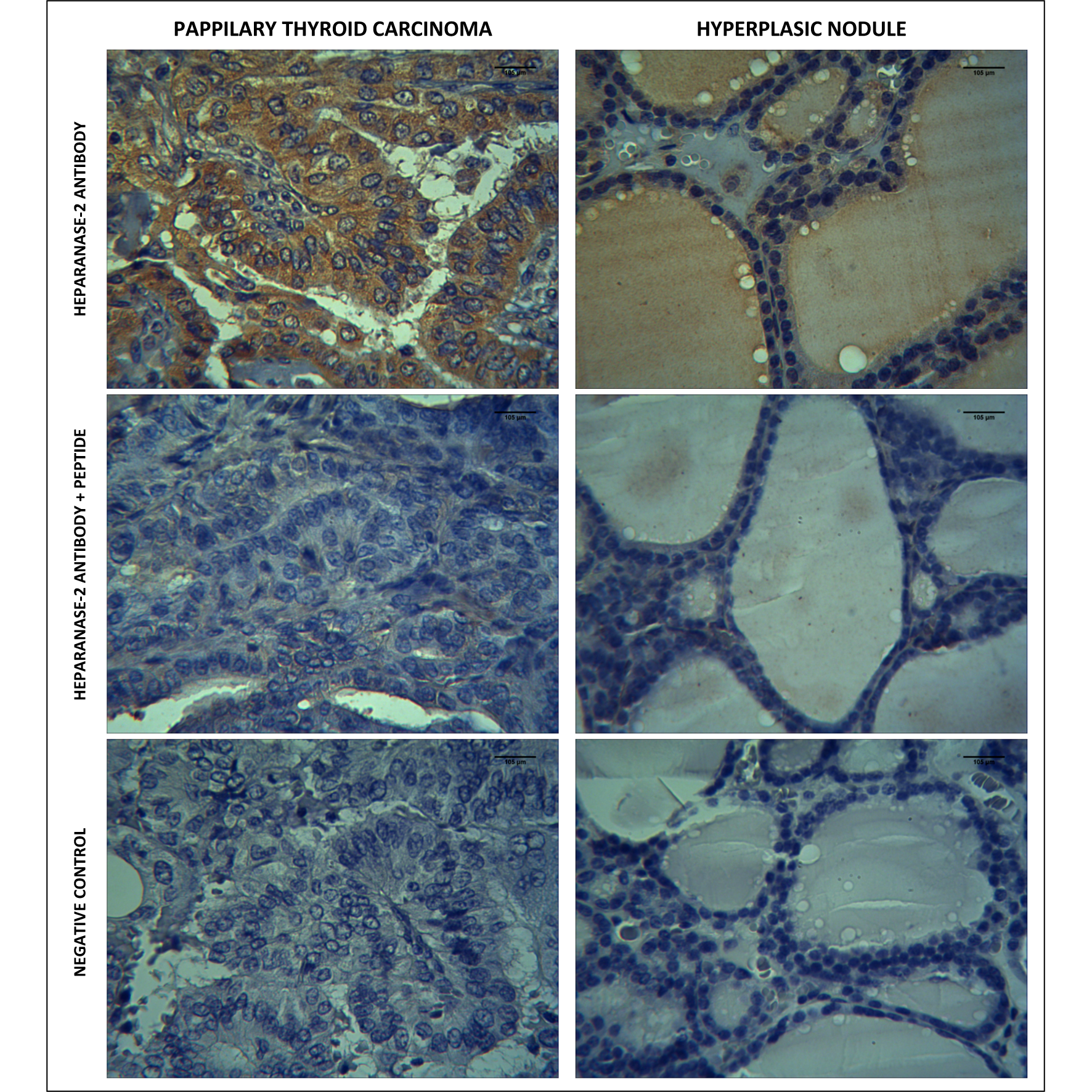

Supplement: S1 Fig — Heparanase-2 antibody, HPA2 C-17 (sc14900; Santa Cruz®, Biotechnology, CA, USA) was used at a final concentration of 2 μg/mL; Heparanase-2 antibody + peptide, Blocking assay combining the antibody HPA2 C-17 at 2 μg/mL with five-fold excess (10 μg/mL) of blocking peptide (sc14900P; Santa Cruz®, Biotechnology, CA, USA), incubated overnight at 4°C, as suggested by manufacturer's instructions, followed by immunohistochemistry assay described in methods. Negative control, immunohistochemistry assay was performed in the absence of primary antibody. There was no immunostaining in follicular cells of pappilary thyroid carcinoma and also in colloid of a hyperplasic nodule using heparanase-2 antibody. The images were obtained using light microscopy. The bars represent 105 μm. (TIF) [file pone.0141139.s001.tif]
